# Supplementary material for: Probing the structure of water in individual living cells
Source: Nat Commun. 2024 Jun 20;15:5271. doi: 10.1038/s41467-024-49404-9 (PMC11190263; doi:10.1038/s41467-024-49404-9)
Supplement: Supplementary file 1 — Supplementary Information [file 41467_2024_49404_MOESM1_ESM.pdf]

Supplementary Information

Probing the Structure of Water in Living Cells

Xiaoqi Lang, Lixue Shi, Zhilun Zhao, Wei Min*

Department of Chemistry, Columbia University

Table of contents:

	Page
Supplemental Discussion (1-5)	3-6
Fig. S1: Time series analysis on Raman spectral acquisition	7
Fig. S2: Vacuum dehydrated cell spectra	8
Fig. S3: Protein contribution in dehydrated cell spectra	9
Fig. S4: Vibrational solvatochromism study of N-Methylacetamide (NMA)	10
Fig. S5: Spectral features of amide I and III peaks under dehydration and denaturation	11
Fig. S6: Spectral reconstruction using simulated dehydrated cell spectra and bulk water	12
Fig. S7: Consistency and stability of Raman spectral measurement of bulk water	13
Fig. S8: Overview of Raman-MCR analysis workflow	14
Fig. S9: Tests of Raman-MCR analysis with spectrally-altered inputs	15
Fig. S10: Raman-MCR analysis under external osmosis perturbations	16
Fig. S11: Correlation between TOP (q) and the average OH frequency of pure water	17
Fig. S12: Spectral comparison of dangling O-H bonds	17
Fig. S13: Estimation of protein concentration in living HeLa cells *in situ*	18
Fig. S14: Relative solvent-accessible surface area of monomeric proteins	19
Fig. S15: Biointerfacial water spectra of individual single cells of different cell types	20
Table S1: Intracellular concentrations of ions	21
Table S2: Vibrational frequency of NMA in neat solvent	21

Supplementary Discussion 1:
Protein N-H (mainly backbone N-H) is the major contributor to solute background.

1. Expectation from literature
Ref[1] calculated the intracellular concentration of N-H and non-water-OH groups based on the known constitution of *E.coli* and yeast and spores, listed as follows: (unit: mol)

	Backbone NH	Side-Chain NH	Other NH	Other O-H
E.coli	$1.4 * 10^{-15}$	$6.9 * 10^{-16}$	$4.1 * 10^{-16}$	$2.5 * 10^{-16}$
Yeast	$7.6 * 10^{-14}$	$2.0 * 10^{-14}$	$1.3 * 10^{-14}$	$3.2 * 10^{-14}$
Bacterial Spores	$2.9 * 10^{-15}$	$5.0 * 10^{-16}$	$1.6 * 10^{-18}$	$6.1 * 10^{-16}$

The N-H groups on the protein peptide backbone are found to be about 3~4 times more abundant than N-H groups on protein side-chain amino acids and other N-H /O-H groups (such as in DNA/RNA, carbohydrates). This makes sense, as each amino acid would correspond to one N-H group on the peptide backbone but only a small fraction of amino acids actually carry N-H groups in their side chains. Additionally, Ref[2] lists that proteins constitute about 75% of dry weight of Hela cells. Together, these literature evidences lead us to expect N-H vibrations of protein (especially on backbone) as the major contributor to the intracellular solute spectra in the O-H stretching region.

2. Experimental evidence: Comparison between intracellular spectrum and pure protein model
To quantify the protein contribution to the ensemble solute spectra (dehydrated cell spectra, Fig. S3a), we can calculate the ratio between i_{3300} and i_{2930} , and compared with standard protein (BSA). Since both lipid and protein have contributions to 2930 cm^{-1} peak in the cell spectrum, we need to extract the pure protein signal of 2930 cm^{-1} ($I_{P\ 2930}$) from the measured total intensity (i_{2930}) using an unmixing algorithm:

$$\begin{bmatrix} i_{2853} \\ i_{2930} \end{bmatrix} = \begin{bmatrix} I_{L\ 2853} & I_{P\ 2853} \\ I_{L\ 2930} & I_{P\ 2930} \end{bmatrix} \begin{bmatrix} C_L \\ C_P \end{bmatrix} \qquad \text{Eq. (S1)}$$

where C_L and C_P are concentrations of lipid and protein in Raman spectra, can be calculated as

$$C_L = \frac{i_{2930} \cdot I_{P\ 2853} - i_{2853} \cdot I_{P\ 2930}}{I_{L\ 2930} \cdot I_{P\ 2853} - I_{L\ 2853} \cdot I_{P\ 2930}}$$

$$C_P = \frac{i_{2853} \cdot I_{L\ 2930} - i_{2930} \cdot I_{L\ 2853}}{I_{L\ 2930} \cdot I_{P\ 2853} - I_{L\ 2853} \cdot I_{P\ 2930}}$$

Here, we used glycerol trioleate (GTO) and Bovine serum albumin (BSA) as the model compounds for lipids and proteins, respectively. After unmixing, we obtained $\frac{\boldsymbol{I_{P\ 2930}}}{\boldsymbol{i_{2930}}}$ = 86.7%, then we calculated the ratio $\frac{\boldsymbol{i_{3300}}}{\boldsymbol{I_{P\ 2930}}} = \frac{0.13}{1 * 86.7\%}$ = 0.15. This intracellular value is consistent with the ratio obtained from pure BSA protein *in vitro*: $\frac{\boldsymbol{I_{P\ 3300}}}{\boldsymbol{I_{P\ 2930}}} = \frac{0.15}{1}$ = 0.15 (Fig. S3b). This consistency indicates that N-H groups in proteins (rather than other N-H or O-H carrying biomolecules inside cells such as carbohydrates, DNA/RNA or residual water) are the main contributor to our measured intracellular solute background after vacuum dehydration in O-H spectral window (3100 - 3800

cm^{-1}). Otherwise, one would have observed a significantly higher ratio for intracellular spectrum than that of pure protein *in vitro*.

Supplementary Discussion 2:

Estimate of spectral shifts of vacuum-dehydrated cell spectra.

In this study we have proposed to measure intracellular solute spectra in an isothermal vacuum dehydrated condition. Despite the simplicity, further justification is needed on how much the resulting spectrum truly represents the N-H features in *living* cells. It is natural to raise the concern of whether conformation change of proteins during vacuum dehydration will distort the spectrum, such as inducing spectral shift or amplitude change compared to the hydrated condition. To resolve this concern, we leverage the concept of proximity probe, the carbonyl group (C=O) in the amide unit, to quantify N-H response to environmental change.

In the Vibrational Stark Spectroscopy[3], Stark tuning rate $|\Delta\vec{\mu}|$ is used to quantify and calibrate how much of a spectral shift (in cm^{-1}) is expected for 1 MV/cm of an electric field projected onto the bond axis. . For common functional group in organic molecules, Stark tuning rates are measured to be around 0.5-2 cm^{-1}/(MV/cm) [3]. Specifically, the Stark tuning rate for C=O in NMA, a model compound for amide, has been recently determined as 0.67 cm^{-1}/(MV/cm) [4].

To quantitatively gauge the N-H response in protein backbones, we sought to measure the Stark tuning rate of N-H bonds in NMA and benchmark it to that of C=O bonds. Fig. 2d depicts the result of vibrational solvatochromism of NMA dissolved in different solvents (from THF to DMSO) with various polarities. The peak shifts of N-H stretching frequency follow a similar trend to the peak shifts of C=O bond in NMA. This is consistent with our picture that similar Onsager reaction fields are experienced by these two bonds due to the physical proximity and parallel bond axis. In this picture, the slope of Fig. 2d will characterize the ratio of Stark tuning rates between N-H group and C=O group inside NMA. Quantitatively, N-H vibration is expected to respond to environmental change with a 2.8 larger magnitude than C=O bonds, and we estimate its Stark tuning rate to be around 0.67x2.8 = 1.8 cm^{-1}/(MV/cm), which still falls within the common range of 0.5-2 cm^{-1}/(MV/cm). Finally, given a 1.4 cm^{-1} blue-shift of C=O vibration between dehydrated cells and live cells observed in Fig. 2c, the estimated shift for N-H vibration in amide is approximated to 1.4x2.8 = 4 cm^{-1}. Butylamine, a model compound we chose for N-H group in protein side chains, shows a similar behavior of vibrational solvatochromism (Fig. 2e). The same proximity probe concept can also be applied to BSA model protein, which generates a comparable result (Fig. 2f).

Supplementary Discussion 3:

Reconstructing single-cell water spectrum with two components

With the well-defined dehydrated cell spectra, we try to reconstitute the intracellular water spectrum by adding a varying fraction ($\boldsymbol{x}$) of bulk water spectra and a scaled ($\boldsymbol{y}$) dehydrated cell profile. Mathematically, we define the following function to evaluate the spectral reconstruction.

$$\text{Reconstruction ratio} = \frac{\int_{\omega_1}^{\omega_2} |\, \boldsymbol{x} * Bulk\ water\ Spectra + \boldsymbol{y} * Dehydrated\ Cell\ Spectra \,|}{\int_{\omega_1}^{\omega_2} |\, Live\ Cell\ Spectra \,|} \quad \text{Eq. (S2)}$$

Here, we only consider the O-H spectral window from ω_1 = 3150 cm^{-1} to ω_2 = 3800 cm^{-1}.
x = 0 ~ 1, and y is determined by the C-H peak intensity at 2930 cm^{-1} using the following equation.

$$I_{2930\,(Live\ Cell)} = x * I_{2930\,(Bulk\ water)} + y * I_{2930\,(Dehydrated\ Cell)} \quad \text{Eq. (S3)}$$

Based on these two equations, the reconstruction ratio as the function of pure water weight ($\boldsymbol{x}$) is plotted in Fig. 3a. However, the ratio never reaches 100%, indicating the measured live-cell spectrum in the O-H region cannot be accounted for by just the two components.

Supplementary Discussion 4:
Determination of parameters for whole-cell water model

To model the biointerfacial water, the protein concentration inside live cell needs to be determined appropriately. Experimentally, we measured the Raman spectra aqueous solutions (50 mg/ml to 400 mg/ml) of BSA or lysozyme (Fig. S13 a, c). In order to calibrate protein concentration with the corresponding Raman intensity of 2930 cm^{-1} peak, the O-H contribution of 2930 cm^{-1} peak needs to be removed in the data analysis (Fig. S13 b, d). Fig. S12e illustrates the fitting curve, which enables us to estimate protein concentration in-vivo from Raman intensity measured at the same power and acquisition time. For each cellular spectrum, since both lipid and protein have contribution to 2930 cm^{-1} peak, we first extract the pure protein signal of 2930 cm^{-1} ($I_{P\ 2930}$) from the measured total intensity (i_{2930}) using an unmixing algorithm (Eq. S1). Here, we used glycerol trioleate (GTO) and Bovine serum albumin (BSA) as the model compounds for lipids and proteins (Fig. S13f). Given $I_{P\ 2930}$, we then can obtain the total protein concentration from the determined curve (Fig. S13e), computed to roughly 100 mg/ml.

The average molecular weight of a human protein is 53 kD. Together with experimentally determined 100 mg/ml for intracellular proteins, we can estimate an average protein concentration of 1.87 mM in HeLa cells. This is very close to the reported 1.6 mM by proteomics[5]. Cautiously, not all proteins are water accessible. Among the three major protein types, globular proteins are water-soluble and expected to be the major contributor, whereas membrane and fibrous proteins are structurally buried or form large structure with low water solubility (hence less interface area). Considering the composition weight of membrane protein (1/3) and cytoskeleton (1/4) [6, 7], the globular protein can roughly be ½ of the total protein (i.e., ~ 0.94 mM). Hence, *N*, the average number of interfacial water molecules associated with one globular protein, can be calculated as the concentration ratio (about 1,500) between biointerfacial water (1.4 M) and globular protein (0.94 mM).

The solvent-accessible surface area (SASA) is approximately 18,000 $Å^2$ for an average human protein of 53 kD, extrapolated from the reported empirical power law relation between SASA and molecular weight of proteins (Fig. S14)[8].

Supplemental Discussion 5:
Spectral Analysis Methods

Raman-MCR spectroscopy, essentially a differential spectroscopy technique, are used to quantitatively retrieve the spectra of water perturbed by ions/solutes (termed as solvation/hydration shell)[9, 10]. This approach treats the solution spectrum as a linear combination of three spectral components: (1) intrinsic vibration response of the solute/ion (if any). (2) spectrum of water that is perturbed by the ion/solute. (3) spectrum of unperturbed water that is equivalent to the spectrum of the bulk water. Component (1) and (2) are combinedly treated as

solute-correlated (SC) spectrum or the hydration/solvation shell spectrum. To retrieve SC information, the question becomes how much bulk water spectrum [reference spectrum, $R(\tilde{v})$] shall be subtracted from experimentally measured solution spectrum ($S(\tilde{v})$).

$$SC\ (\tilde{v}) = S(\tilde{v}) - f * R(\tilde{v}) \qquad \text{Eq. (S4)}$$

Two-component Raman-MCR analysis determines solute-affected water spectra $SC\ (\tilde{v})$ based on area-normalized minimum area criterion. It involves the systematic subtraction of maximum fraction (f) of $R(\tilde{v})$ from $S(\tilde{v})$. The subtraction is continued until there appears a *spectral point reaches zero signal* within the vibration resonance of O-H stretch (~3000 – 3700 cm^{-1}).

$$SC\ (\tilde{v}) = S(\tilde{v}) - f_{max} * R(\tilde{v}) \rightarrow nonnegative\ minimum\ area$$

Our approach represents an adaption and expansion of the original two-component Raman-MCR technique, specifically tailored for the context of live cells. In our model, the water spectra within live cells are considered as the linear combination of three distinct components:

1. Intrinsic vibration response of the solute, corresponding to the **dehydrated state of cell**.
2. Spectrum of significantly perturbed water. We refer to this as **bio-interfacial water**, highlighting its altered spectral feature due to close interactions with cellular components.
3. Spectrum of unperturbed water that is equivalent to the spectrum of the **bulk water**.

$$BioWater(\tilde{v}) = LiveCell(\tilde{v}) - x * Ref(\tilde{v}) - y * DehyrCell(\tilde{v}) \rightarrow \text{Non-negative Minimum Area}$$

Eq. (S5)

$$y = \frac{LiveCell_{2930} - x * Ref_{2930}}{DehydrCell_{2930}} \rightarrow \text{Mass Conservation Restriction (at 2930 cm}^{-1}\text{)}$$

Eq. (S6)

In our approach, we apply simultaneous component fitting during subtraction optimization and visualize the differential results in the MCR residual map (Fig. S8). We adapted the core principles of MCR analysis to isolate the vibrational responses of "most perturbed water" by computing the non-negative minimum area of the spectrum. However, we recognized that merely achieving a single point of zero in the spectrum is not always sufficient or indicative of optimal results. Thus, it is remarkably significant in our algorithm because at that particular point, the difference spectrum from 3054 cm^{-1} to 3150 cm^{-1} simultaneously reaches the local minimum. This collective behavior not only guarantees the non-negative minimum-area condition to be fulfilled, as emphasized in Raman-MCR; but serves as an internal check to suggest the existence of an intrinsic spectrum. Subsequently, the bio-interfacial water spectrum is fitted with a minimum number of Gaussian or product of Gaussian to resolve the spectral components (Fig.4 c-d). In our analysis, the "differential" and "curve fitting" analyses were carried out sequentially.

Supplemental Figures

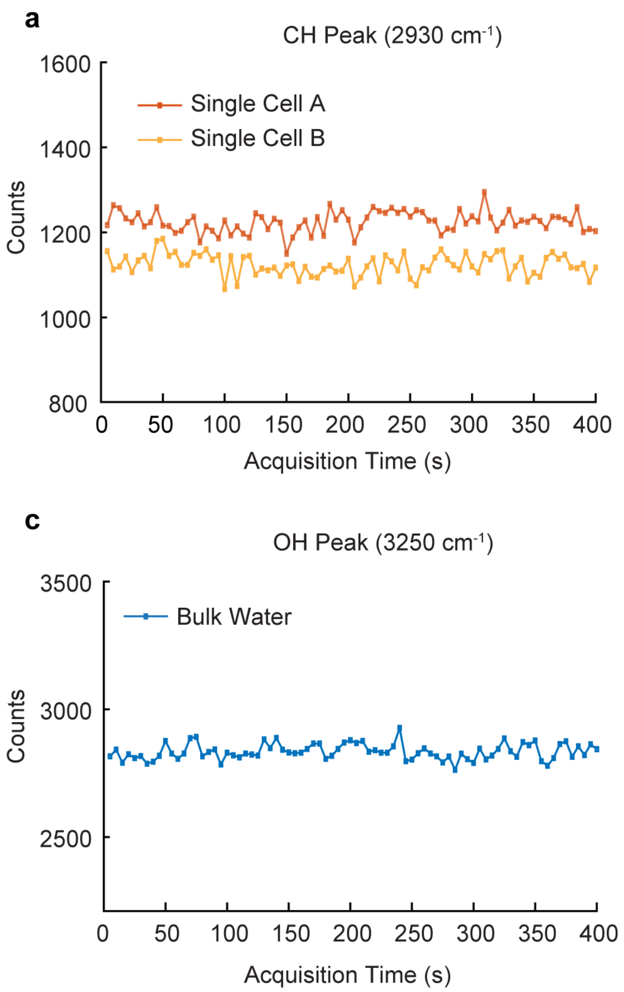

Fig. S1. Time series analysis on Raman spectral acquisition. Single cell and bulk water spectra were recorded over 400s using a home-built Raman spectroscopy. The laser power on sample was 35 mW, and the exposure time for CCD camera was 5s. The intensities of two selected Raman peaks were plotted for cells (a-b) and bulk water (c-d)

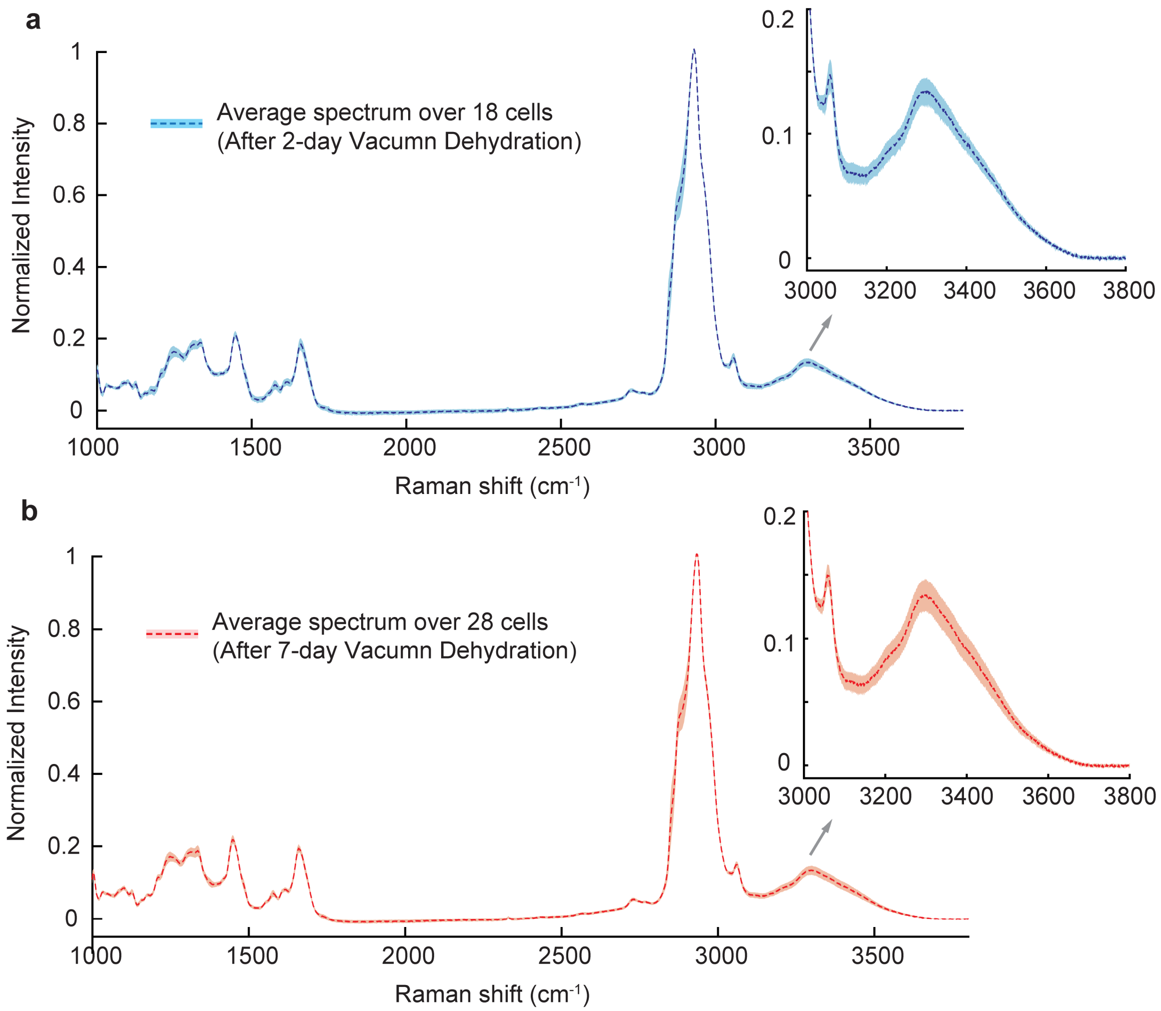

Fig. S2. Vacuum dehydrated cell spectra. Averaged Raman spectrum over multiple vacuum-dehydrated cells after 2-day (**a**) and 7-day dehydration (**b**). The shaded area indicates the standard deviation of the Raman spectrum from different single cells. The averaged spectra are also shown in **Fig. 2a** with good consistency. The 7-day dehydrated spectrum in **b** are used to represent the solute background in the subsequent spectral analysis.

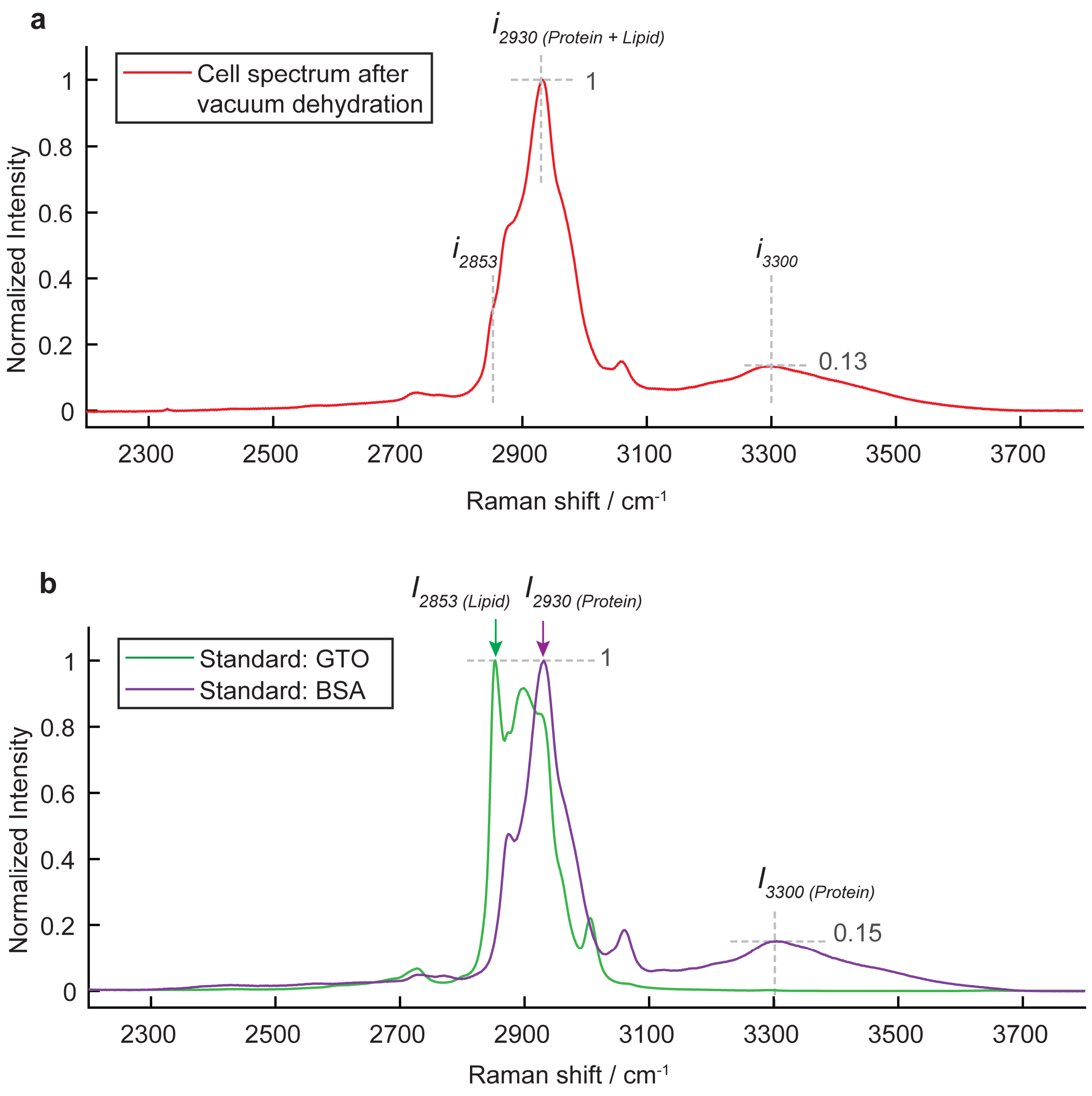

Fig. S3. Protein contribution in dehydrated cell spectra. (a) Averaged cell spectrum after 7-day vacuum dehydration. (b) Raman spectra of model compounds glycerol trioleate (GTO) and BSA (in the form of a dry film). Details in Supplemental Discussion 1.

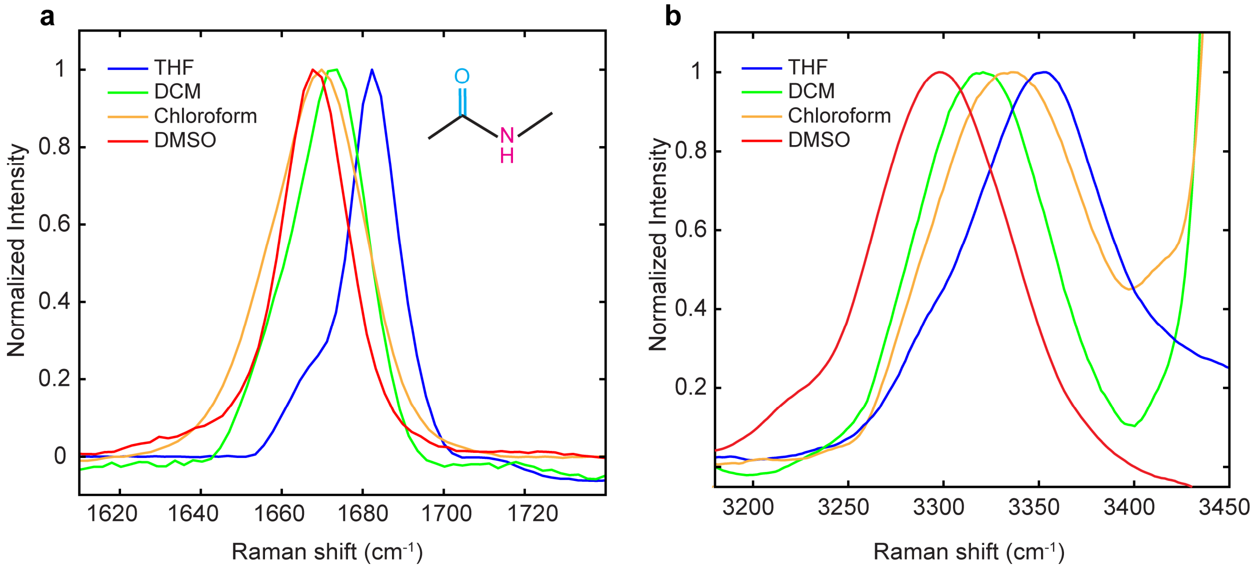

Fig. S4. **Vibrational solvatochromism study of N-Methylacetamide (NMA)**. Raman spectra of the model molecule NMA in different solvents in the C=O region (a) and the N-H region (b). Details of vibrational frequency are listed in Table S2.

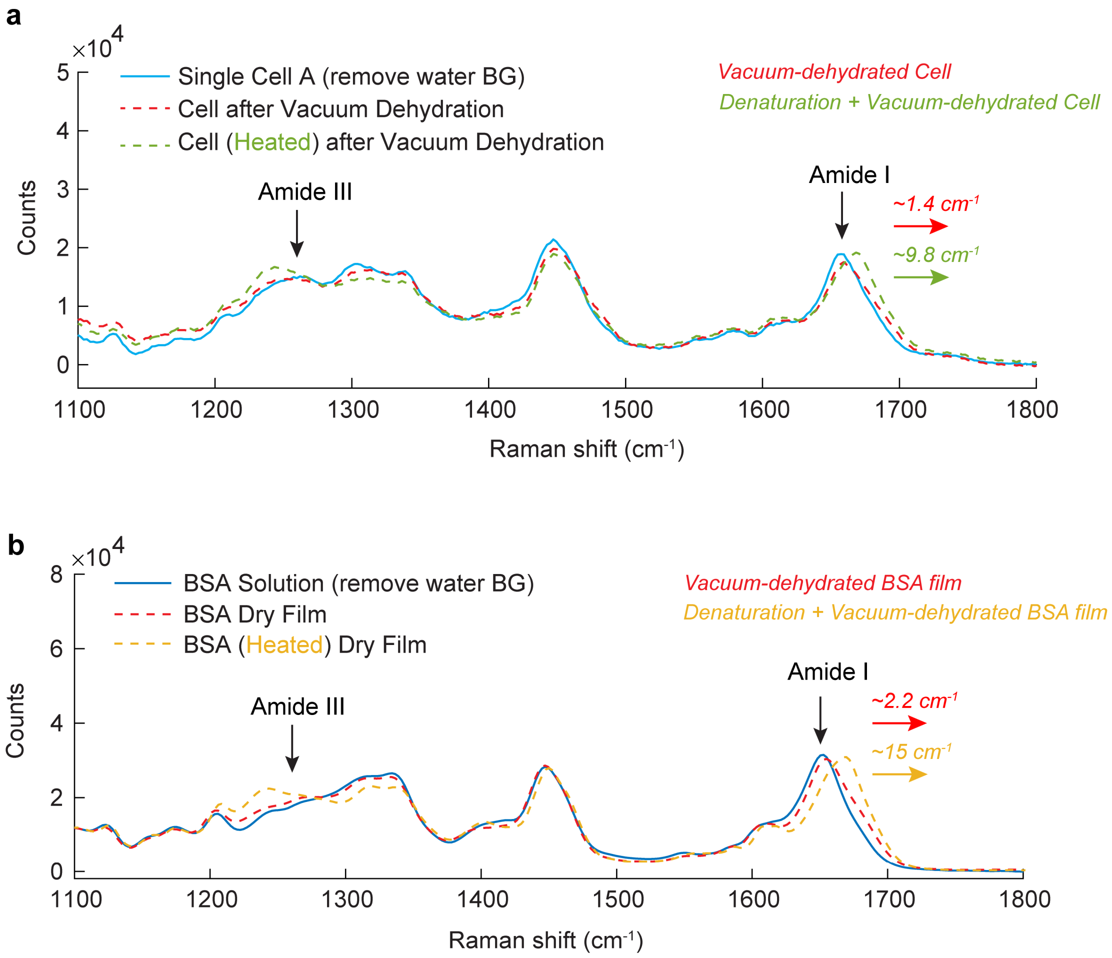

Fig. S5. Spectral features of amide I and III peaks under dehydration and denaturation. (a) Raman spectra of a single living HeLa cell (blue) and 7-day isothermal vacuum dehydrated HeLa cells (red dash line), and HeLa cells heated first followed by dehydration (green dash line). The latter sample was prepared by first heating the living cell dish to 80 °C for 30 mins, then cooling down to R.T. for normal 7-day vacuum dehydration. It shows a blue-shift about 9.8 cm^{-1} of amide I peak after this treatment of denaturation. **(b)** Raman spectra of solvated BSA (in deionized water), BSA dry film and heated BSA dry film. The latter sample was prepared by first heating BSA solution to 80 °C for 30 mins to introduce irreversible protein denaturation. Then the solution is cooled down to R.T. to make a thin dry film on glass. It shows a blue-shift about 15 cm^{-1} of amide I peak after this denaturation treatment. Clearly, the isothermal vacuum dehydration only causes a gentle perturbation to protein conformation in cells (**a**) and in pure BSA (**b**) compared to the harsh denaturation process of heating.

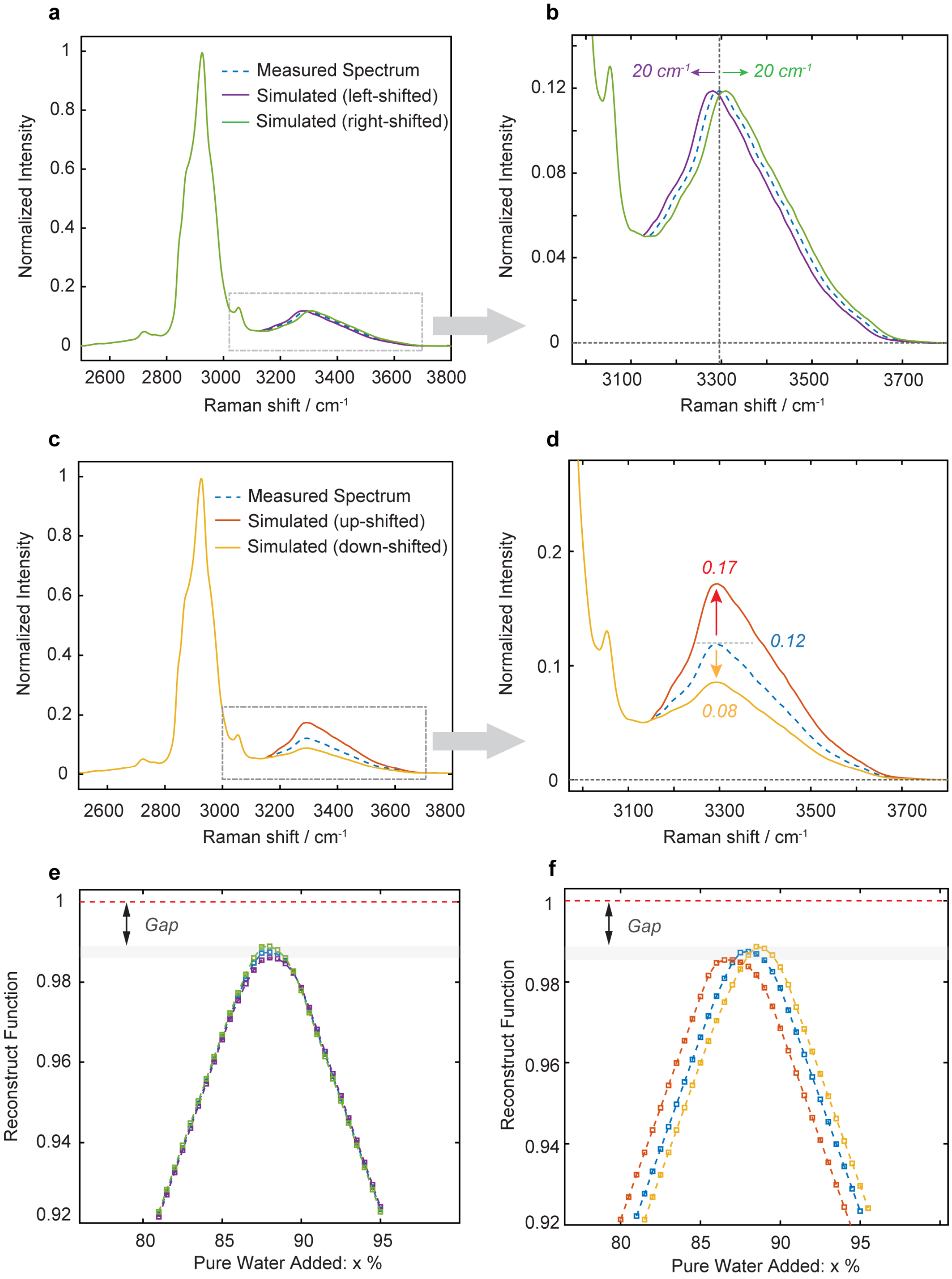

Fig. S6. Spectral reconstruction using simulated dehydrated cell spectra and bulk water. (a-d) Simulated dehydrated cell spectra with altered N-H peak: left-shifted 20 cm^{-1} (purple line), right-shifted 20 cm^{-1} (green line) and amplitude changes (red and yellow line). Experimentally measured dehydrated cell spectra (blue dash line). **(e-f)** Reconstruction functions using simulated dehydrated cell spectra. In all these cases, reconstructions cannot be completed with only two components.

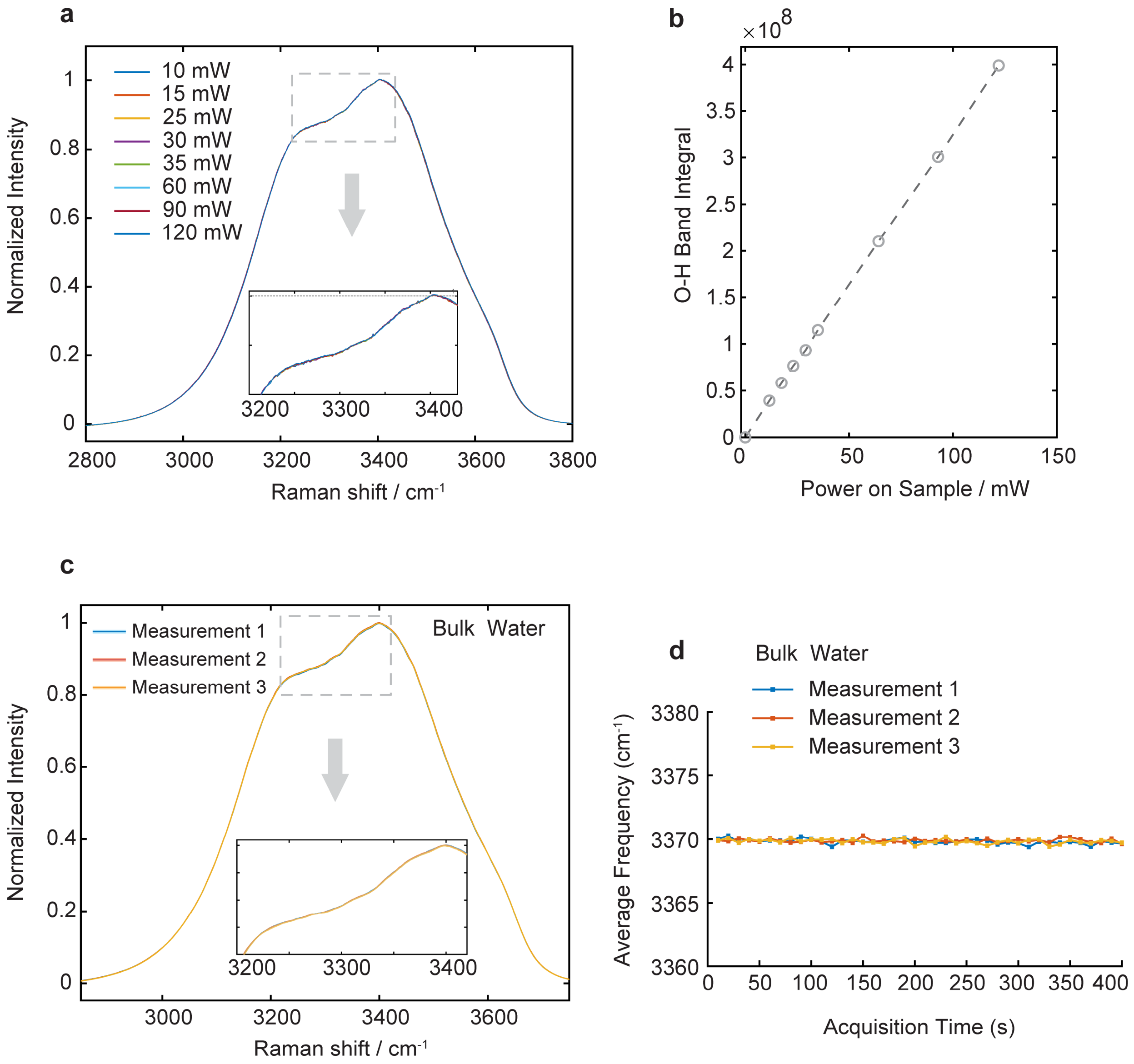

Fig. S7. Consistency and stability of Raman spectral measurement of bulk water. (a) Raman spectra of pure water under different laser powers from 10 mW to 120 mW on sample. **(b)** Intensity of O-H spectra as a function of laser power on water sample. **(c)** Raman spectra of pure water spectra measured during live cell experiments. The averaged frequency is plotted in **(d)**.

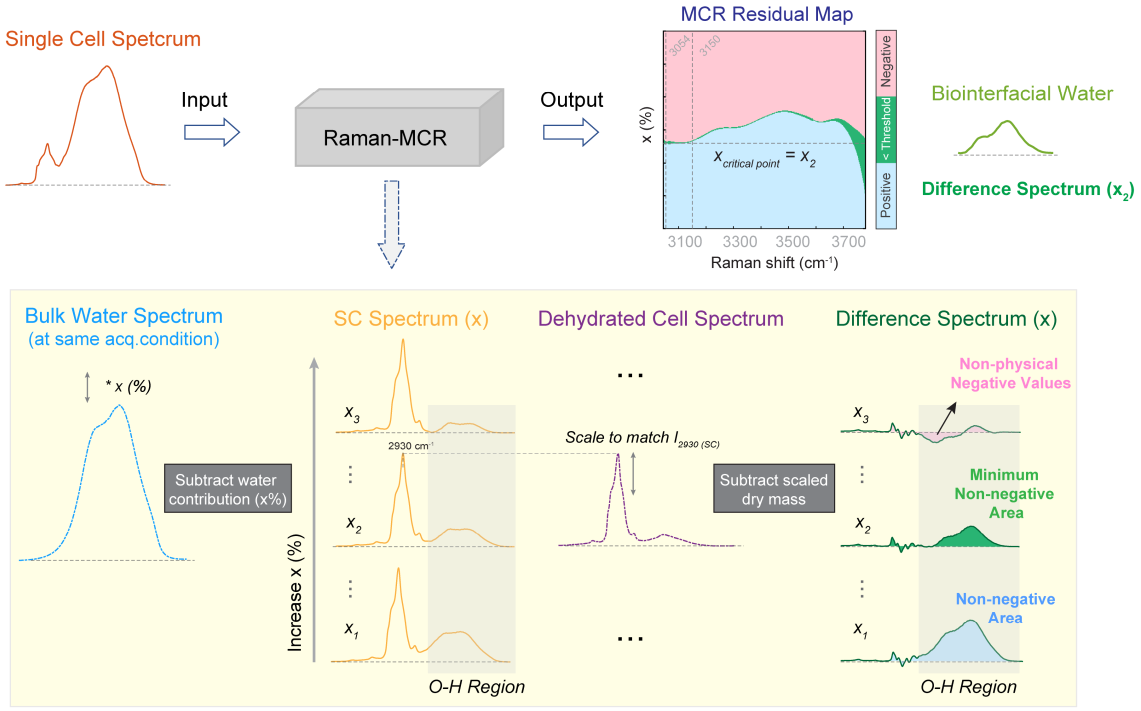

Fig. S8. Overview of Raman-MCR analysis workflow. Input: single cell Raman spectrum and bulk water Raman spectrum at same acquisition condition. Output: MCR residual map and difference spectrum (bio-interfacial water spectrum). The process involves two main steps: (1) *Solvent contribution removal*: A variable fraction (x%) of bulk water spectrum is subtracted from the single cell spectrum to produce Solute-correlated (SC) spectra as a function of (x). (2) *Ensemble solute contribution removal*: Dehydrated cell spectra are normalized based on CH band intensity (2930 cm^{-1}) within SC spectra. These normalized spectra are then subtracted from SC spectra to produce difference spectra for each x. The intensity within difference spectra is divided into three categories: positive, negative, near-zero (below a specified threshold), which are used to construct the MCR residual map for each x value. A critical point is identified at the value of x ($x = x_2$) where the minimum non-negative area is observed.

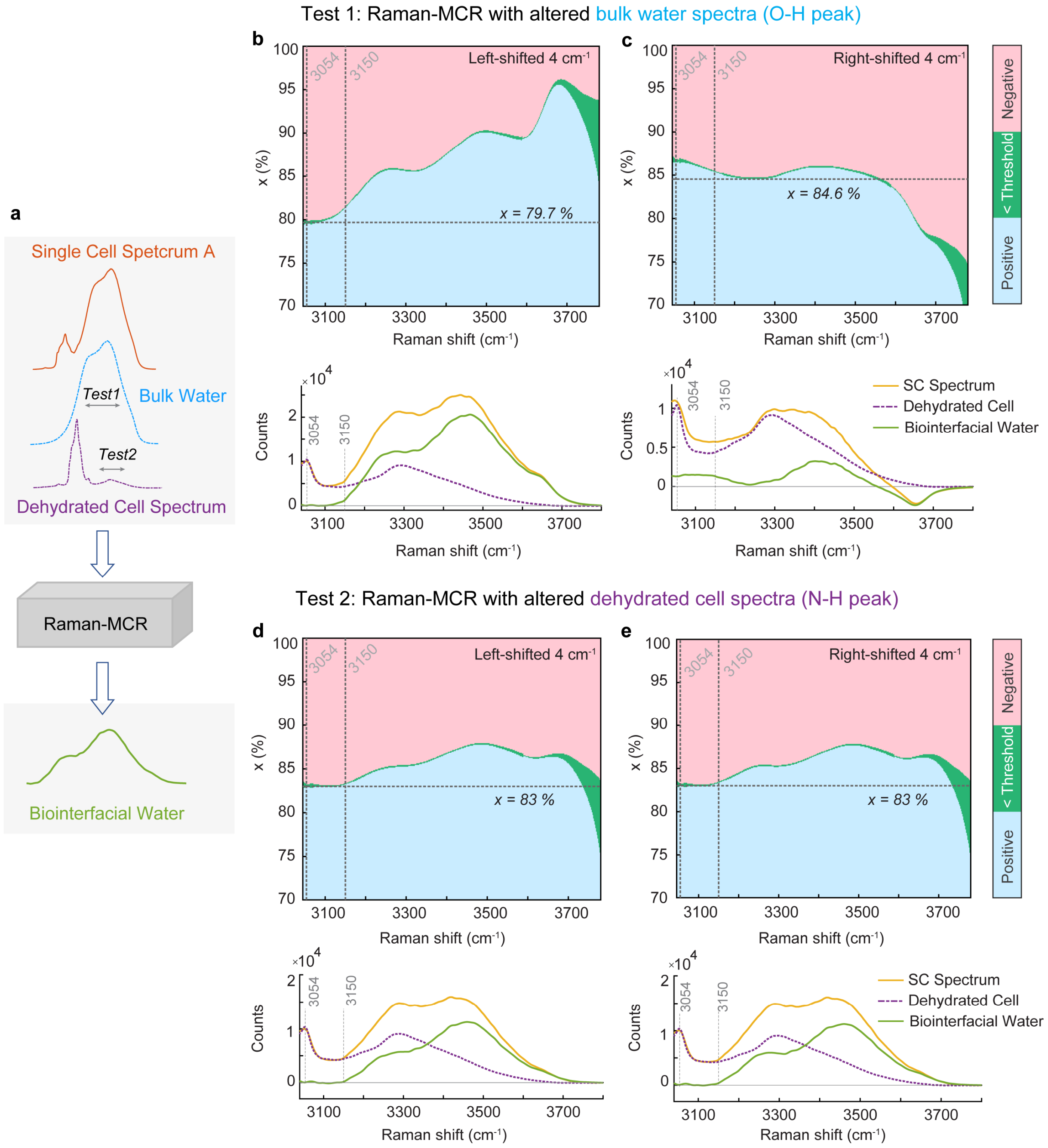

Fig. S9. Tests of Raman-MCR analysis with spectrally-altered inputs. (**a**) Schematics of Raman-MCR testing. As inputs, single live cell spectrum, bulk water spectrum and dehydrated cell spectrum were fed into the Raman-MCR algorithm to generate the output spectrum of biointerfacial water. Tests were performed to examine the sensitivity on different inputs. (**b-c**) Test 1 on shifting the bulk water spectrum (O-H peak) by 4 cm^{-1}. In both cases, the minimum non-negative area condition cannot be fulfilled simultaneously on both sides (3054-3150 cm^{-1} and above 3700 cm^{-1}). Drastic spectral changes are observed compared to Fig. 3f, suggesting the output is sensitive to the quality of the bulk water spectra. (**d-e**) Test 2 on shifting the dehydrated cell spectrum (N-H peak) by 4 cm^{-1}. A similar spectral shape of biointerfacial water can still be obtained (as in Fig. 3f) regardless of the N-H peak shifts.

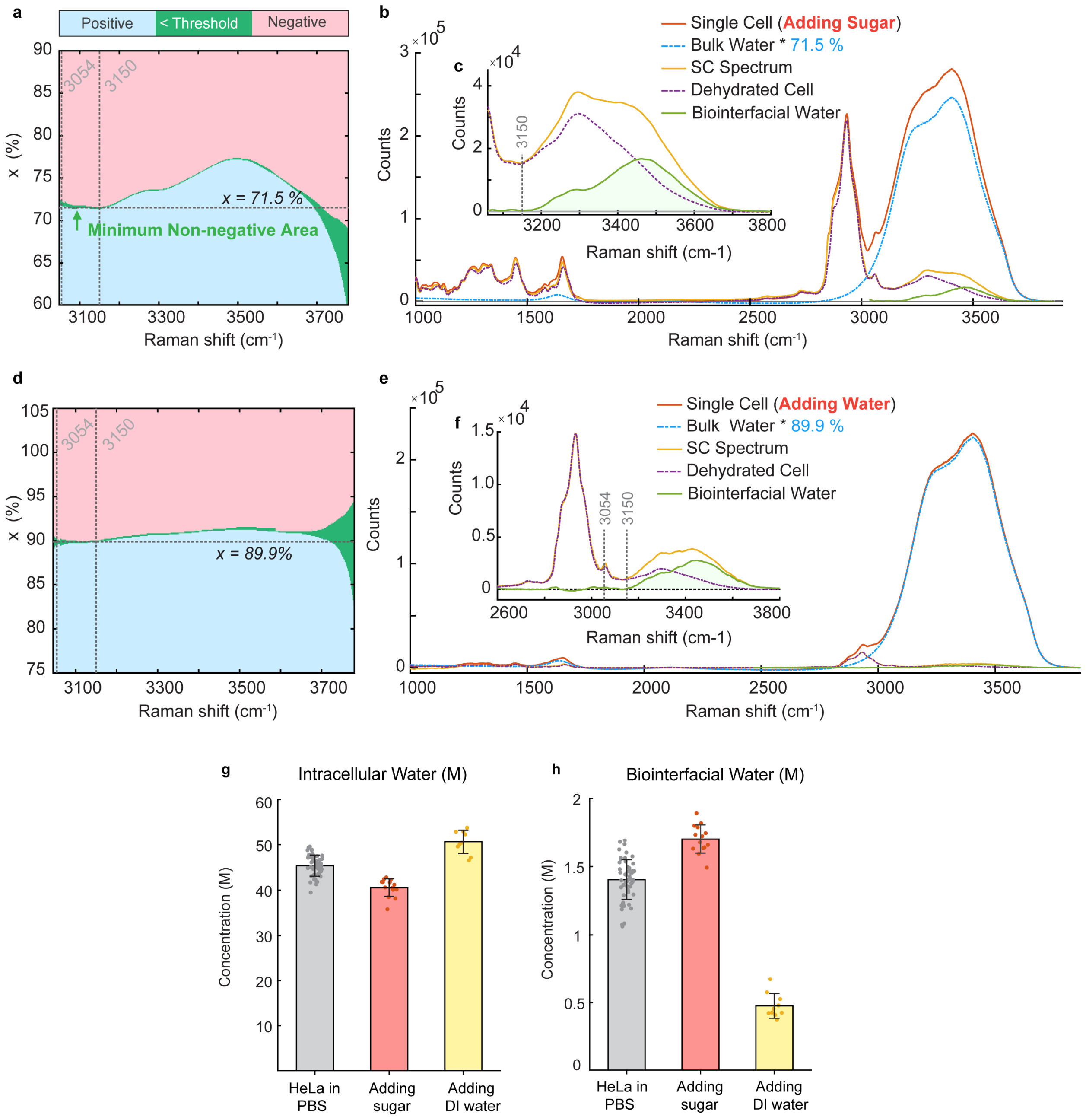

Fig. S10. Raman-MCR analysis under external osmosis perturbations. (**a, d**) Residual Map of Raman-MCR analysis as subtracting varying amounts of bulk water (x%) and scaled dehydrated cell spectra from live-cell water spectra. (**b, e**) Corresponding Raman spectra when subtracting x% of bulk water spectra (blue dash) and scaled dehydrated cell spectra (purple dash) from the live-cell spectra (red line). The final residual is identified as biointerfacial water (characterized as green line). SC Spectra: solute-correlated spectra (yellow line), obtained when only removing bulk water contribution (blue dash) from single cell spectra (red line). (b) 35mW over 400s (c) 30mW over 300s. (**c, f**) zoom-in view of O-H region. (**g, h**) Comparison of three different conditions.

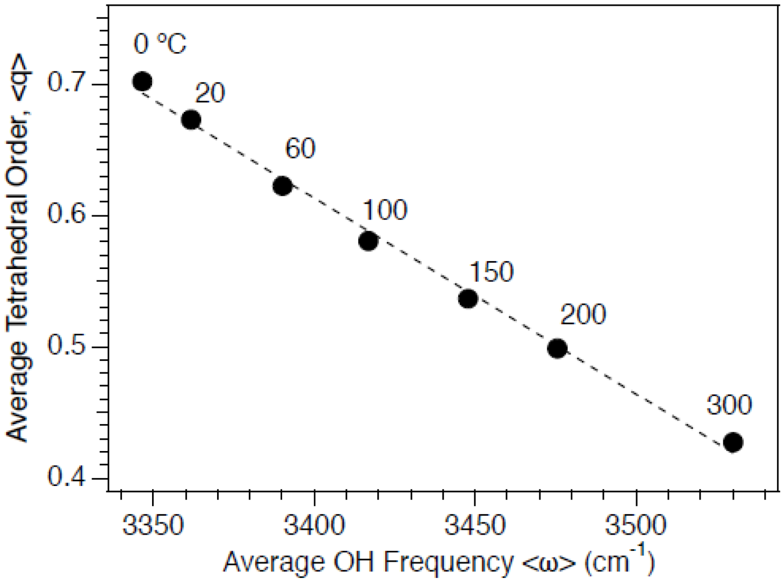

Fig. S11. Correlation between the average tetrahedral order parameter (q) and the average OH frequency of pure water. Adapted from Ref[11]. The approximately linear correlation, with a slope of $dq/d\omega \approx -0.00149$, may be used to estimate the tetrahedrality differences between the biointerfacial water and pure water, from the corresponding experimental OH frequency differences $\Delta q \approx -0.00149 * \Delta\omega$.

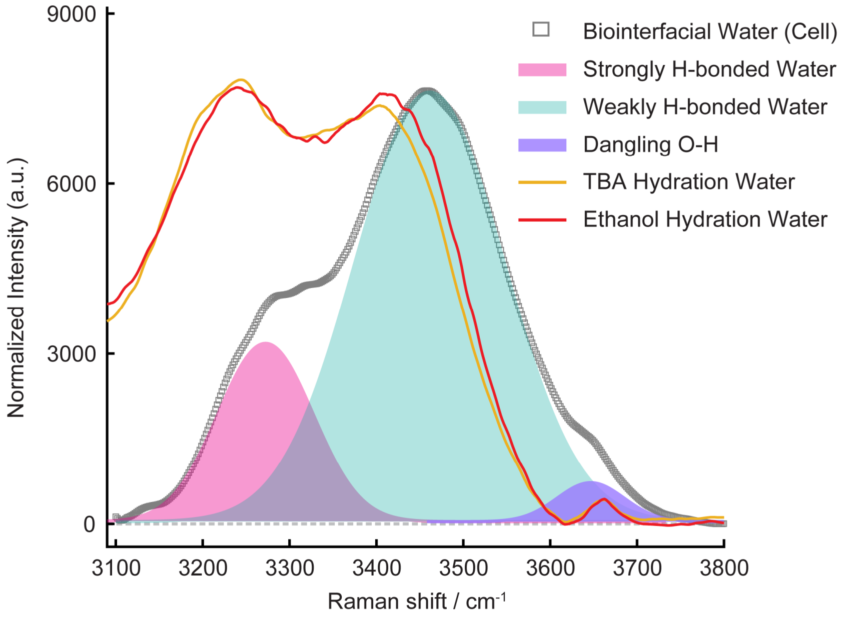

Fig. S12. Spectral comparison of dangling O-H bonds between biointerfacial water and hydration water around small molecules (tert-butyl alcohol and ethanol).

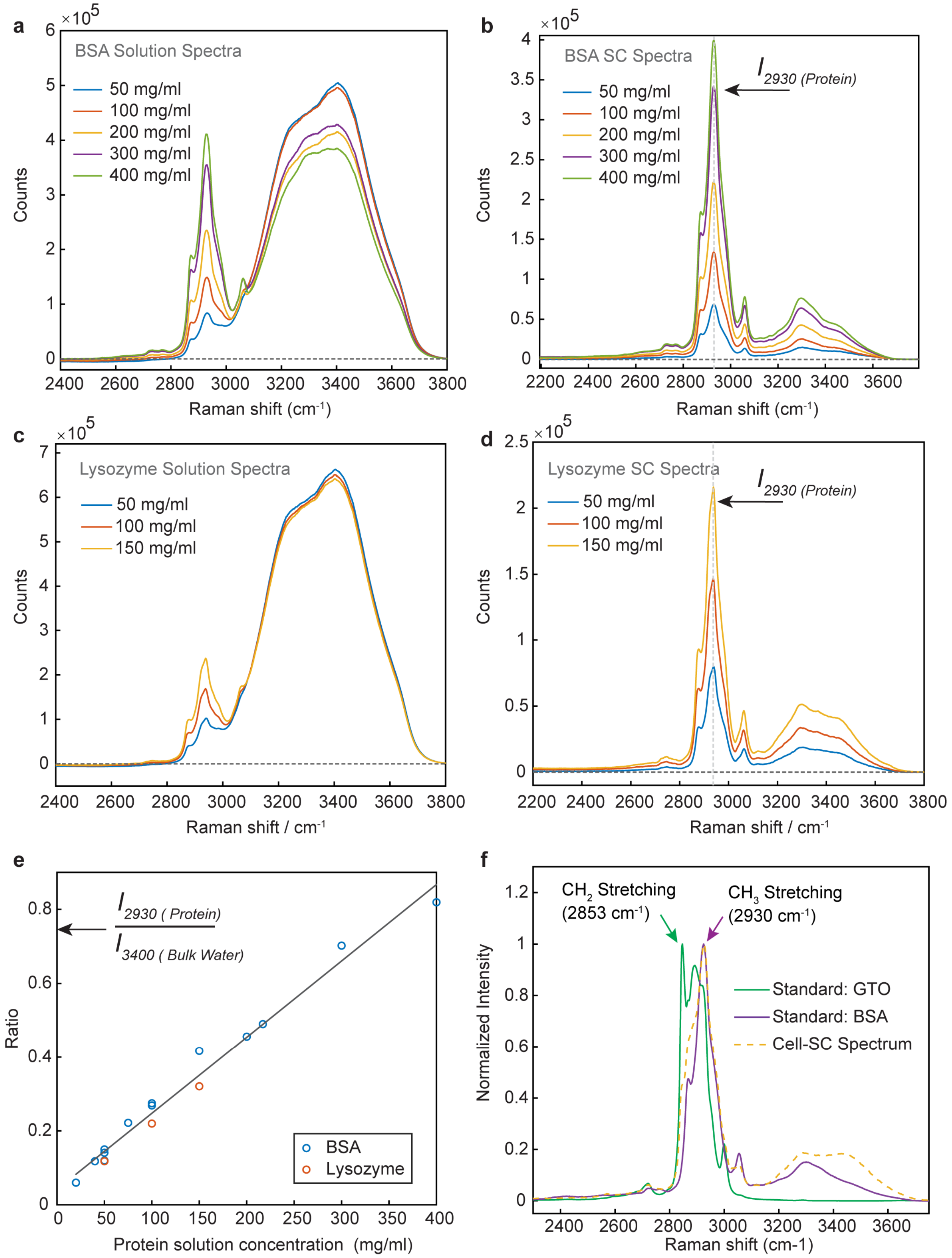
a
BSA Solution Spectra
50 mg/ml
100 mg/ml
200 mg/ml
300 mg/ml
400 mg/ml
Counts
×10^5
Raman shift (cm-1)
b
BSA SC Spectra
I2930 (Protein)
50 mg/ml
100 mg/ml
200 mg/ml
300 mg/ml
400 mg/ml
Counts
×10^5
Raman shift (cm-1)
c
Lysozyme Solution Spectra
50 mg/ml
100 mg/ml
150 mg/ml
Counts
×10^5
Raman shift / cm-1
d
Lysozyme SC Spectra
I2930 (Protein)
50 mg/ml
100 mg/ml
150 mg/ml
Counts
×10^5
Raman shift / cm-1
e
I2930 (Protein) / I3400 (Bulk Water)
BSA
Lysozyme
Ratio
Protein solution concentration (mg/ml)
f
CH2 Stretching (2853 cm-1)
CH3 Stretching (2930 cm-1)
Standard: GTO
Standard: BSA
Cell-SC Spectrum
Normalized Intensity
Raman shift (cm-1)

Fig. S13. Estimation of protein concentration in living HeLa cells *in situ*. (a) Raman spectra of BSA solutions measured at 50 mW over 300 s. **(b)** Solute-correlated spectra of BSA solution after removing pure water contribution (non-negative minimum area) **(c)** Raman spectra of Lysozyme solutions measured at 50 mW over 300 s. **(d)** Solute-correlated spectra of Lysozyme solution after removing pure water contribution (non-negative minimum area). (**e**) Calibration curve of protein concentration using normalized I_{P2930}. (f) Unmixing of protein concentration in single cells based on model compounds glycerol trioleate (GTO) and BSA. Details in *Supplemental Discussion 4*.

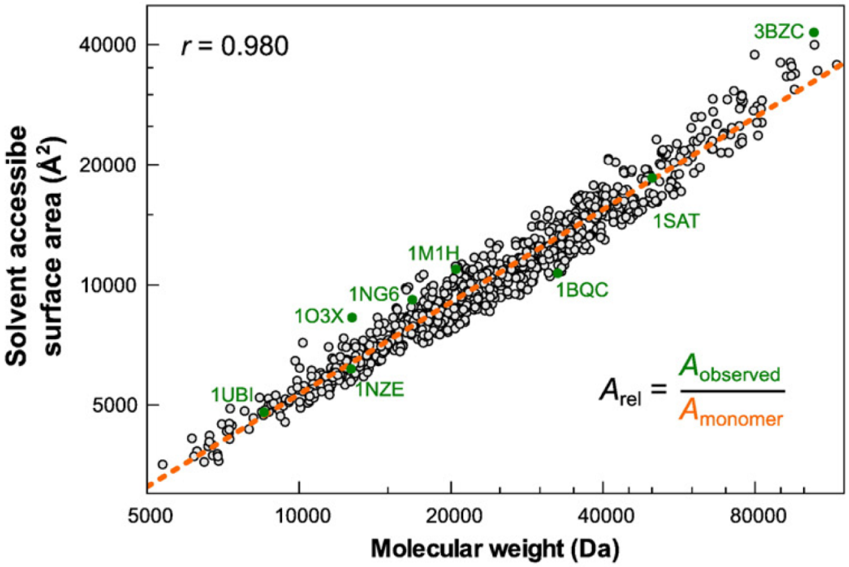

Fig. S14. Relative solvent-accessible surface area of monomeric proteins. Comparison of molecular weight and solvent-accessible surface area values calculated from 907 non-redundant, high-confidence folded monomeric crystal structures. An empirical power law relation was reported. Adopted from Ref [8].

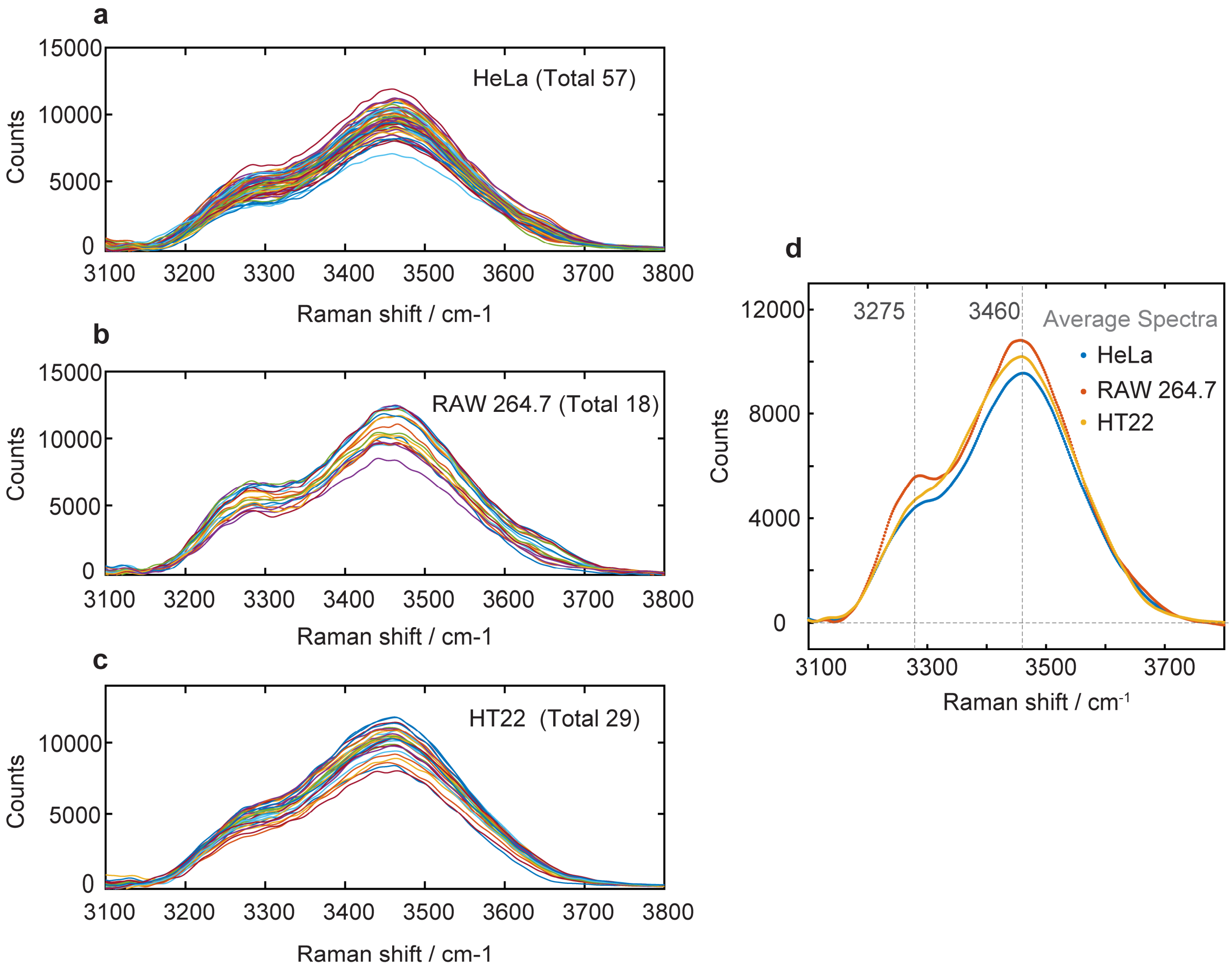

Fig. S15. Biointerfacial water spectra of individual single cells of different cell types. (a) HeLa cells (total number = 57)**. (b)** RAW 264.7 cells (total number = 18)**. (b)** HT22 cells (total number = 29)**. (d)** Averaged biointerfacial water spectra of different cell lines.

Table S1. Intracellular concentrations of ions, adopted from Ref [12]

	**Concentration**
**Na^+**	15 mM
**K^+**	150 mM
**P_i**	40 mM
**Cl^-**	10 mM
**HCO_3^-**	10 mM
**H^+**	10^{-7} M
**Ca^{2+}**	10^{-8} mM
**Mg^{2+}**	5 mM

Table S2: Vibrational frequency of **NMA** in neat solvent

**Solvent**	**C=O / cm^{-1}**	**N-H /cm^{-1}**
THF	1683.5	3354.4
DCM	1673.9	3324.2
Chloroform	1669.7	3333.1
DMSO	1668.1	3299.3

1. M. Tros, L. L. Zheng, J. Hunger, M. Bonn, D. Bonn, G. J. Smits and S. Woutersen, Nat. Commun. **8**, 7 (2017).
2. F. Feijó Delgado, N. Cermak, V. C. Hecht, S. Son, Y. Li, S. M. Knudsen, S. Olcum, J. M. Higgins, J. Chen and W. H. Grover, Plos One **8** (7), e67590 (2013).
3. S. D. Fried and S. G. Boxer, Accounts Chem Res **48** (4), 998-1006 (2015).
4. S. D. E. Fried, C. Zheng, Y. Z. Mao, T. E. Markland and S. G. Boxer, J Phys Chem B **126** (31), 5876-5886 (2022).
5. R. Milo, Bioessays **35** (12), 1050-1055 (2013).

6. M. Beck, A. Schmidt, J. Malmstroem, M. Claassen, A. Ori, A. Szymborska, F. Herzog, O. Rinner, J. Ellenberg and R. Aebersold, Mol Syst Biol **7** (2011).
7. W. Liebermeister, E. Noor, A. Flamholz, D. Davidi, J. Bernhardt and R. Milo, Proc. Natl. Acad. Sci. U. S. A. **111** (23), 8488-8493 (2014).
8. J. A. Marsh, J Mol Biol **425** (17), 3250-3263 (2013).
9. K. R. Fega, A. S. Wilcox and D. Ben-Amotz, Appl Spectrosc **66** (3), 282-288 (2012).
10. N. Ghosh, S. Roy, A. Bandyopadhyay and J. A. Mondal, Liquids **3** (1), 19-39 (2022).
11. X. E. Wu, W. J. Lu, L. M. Streacker, H. S. Ashbaugh and D. Ben-Amotz, Angew Chem Int Edit **57** (46), 15133-15137 (2018).
12. S. Niederer, Plos One **8** (4) (2013).
